# *Origanum vulgare* terpenoids modulate *Myrmica scabrinodis* brain biogenic amines and ant behaviour

**DOI:** 10.1371/journal.pone.0209047

**Published:** 2018-12-26

**Authors:** Giuseppe Mannino, Gholamreza Abdi, Massimo Emilio Maffei, Francesca Barbero

**Affiliations:** Department of Life Sciences and Systems Biology, Innovation Centre, University of Turin, Turin, Italy; Philipps-Universitat Marburg Fachbereich Biologie, GERMANY

## Abstract

Coordinated social behaviour is fundamental for ant ecological success. However, even distantly-related organisms, such as plants, have evolved the ability to manipulate ant collective performances to their own advantage. In the parasitic system encompassing *Maculinea* butterflies, *Myrmica* ants, and *Origanum vulgare* plants, the ant-plant interaction elicits the release of a volatile terpenoid compound (carvacrol) which is used by the gravid butterfly to locate the ideal oviposition site. Here we show that this ant-plant association is maintained by the effect of *O*. *vulgare* terpenoids on ant behaviour and that food plants might gain protection by *Myrmica* ants by chemically manipulating workers to forage in their surroundings. The variation in the locomotor ability of three ant species (*Formica cinerea*, *Tetramorium caespitum*, and *Myrmica scabrinodis*) was studied after treatment with the two major *O*. *vulgare* terpenoid volatile compounds (i.e., carvacrol and thymol). The brain levels of three biogenic amines (dopamine, tyramine and serotonin) were analysed in ants exposed to the *O*. *vulgare* terpenoids by HPLC-ESI-MS/MS. Carvacrol and thymol increased the locomotor activity of all ant species tested, but if blended reduced the movement propensity of *Myrmica scabrinodis*. Dopamine and tyramine production was positively correlated with the worker locomotor activity. In *Myrmica* ants, both brain biogenic ammines were negatively correlated with the aggressive behaviour. Blends of *O*. *vulgare* volatiles affected the locomotor ability while increased the aggressiveness of *Myrmica* workers by altering the aminergic regulation in the ant brains. This behavioural manipulation, might enhance partner fidelity and plant protection. Our findings provide new insights supporting a direct role of plant volatiles in driving behavioural changes in social insects through biogenic amine modulation.

## Introduction

The social organization of ants is based on communication signals which coordinate large numbers of individuals in collective-decision processes, without a centralised control [1]. Although some decisions are individually made, the majority of choices influencing the colony success are performed cooperatively. The decision-making occurs in the selection of the most appropriate food in terms of amount and quality [2], best migration or foraging route [3,4], most suitable place where to build a new nest [5,6] as well as decisions to attack either enemies or competitors [7,8]. This peculiar trait of eusocial organisms, together with the ability to regulate the number of individuals performing specific tasks (division of labour), enhances the colony plasticity to react promptly against biotic and abiotic variations [9,10].

Plant chemical signalling can manipulate and influence the decision-making of ants [11–13]. However, how the manipulation of social activities is achieved is not fully disentangled and the mechanism is likely to vary in distinct systems. A role of biogenic amines in driving behavioural changes of both vertebrates [14,15] and invertebrates [16–19], including ants [20–22], has been demonstrated. Research on ants is still in its infancy, yet some supporting evidence was provided for colonies of the Red Imported Fire Ant, *Solenopsis invicta*, with a change in the nest-mate recognition determined by a decrease in the brain level of octopamine [23], and in *Pheidole* ants, where the depletion in the serotonin content modulates the trail-following behaviour of workers [24].

In spite of their ecological dominance, ants suffer from social parasitism by several invertebrates [1]. *Maculinea* butterflies are outstanding examples of cheaters employing both chemical [25] and acoustical [26] strategies to exploit their *Myrmica* host ants. In this multitrophic system, the ant-butterfly association is obligately parasitic and involves also the species-specific plant, used as the egg-laying site by butterfly females. In the case of *Maculinea arion*, early-instar butterfly larvae feed on *Origanum vulgare*, until they drop on the ground where thanks to chemical mimicry are retrieved by *Myrmica* foragers and taken into the ant nest. Here *M*. *arion* larvae spend the next ten months consuming the resources of the colony and feeding on the ant brood until the adult will eventually emerge from the nest [27].

Recently, it has been demonstrated that the interaction between *Myrmica* ants and *O*. *vulgare* plants leads to an increased production of the plant volatile monoterpene carvacrol [28]. The release of carvacrol is the indirect signal interpreted by *M*. *arion* gravid females to locate the food plant growing in the vicinity of its *Myrmica* host colonies, thus providing its brood with both sequential hosts (i.e., the source of food, shelter and care in the nest) [28,29]. *Myrmica* ants survive longer than other ant species when exposed to carvacrol, by upregulating specific genes coding for detoxifying enzymes [28]. Therefore, it seems that *Myrmica* ants benefit from being resistant to carvacrol by occupying a competitor-free spaces surrounding oregano plants, while parasite larvae increase their chance of encountering ants [28,29].

The mechanism that induces *Myrmica* ants to forage and found their nests in the proximity of oregano plants is still unknown. Here we show that thymol and carvacrol produced by *O*. *vulgare* act on *Myrmica* behaviour by modifying the biogenic amine level in their brain, thus affecting their locomotor activity and the aggressive behaviour. We also tested and compared the effect of these two monoterpenes on other ant species (*Tetramorium caespitum* and *Formica cinerea*) and confirmed that behavioural changes are correlated to variation on terpenoid-modulated biogenic amines levels.

## Materials and methods

### Animal material

Colonies of *Myrmica scabrinodis*, *Formica cinerea* and *Tetramorium caespitum* were collected at the Parco Fluviale Gesso e Stura, North Italy (44°25’N, 7°35E, 440 m), where a previous study on acute exposure of ants to specific *O*. *vulgare* volatiles was made [28]. Ant colonies were collected and reared in plastic boxes (24×24×9 cm). Ants were fed twice a week on a honey and protein diet [30].

Although our field study did not involve endangered or protected species, the Parco Fluviale Gesso e Stura issued the permission to collect ant specimens for each location.

### Chemicals

GC-grade carvacrol and thymol were purchased from Sigma-Aldrich (St. Louis, MO. USA). Pure standards (dopamine, tyramine, 3,4-dihydroxybenzylamine hydrobromide and serotonin) and formic acid were purchased from Merck (Darmstadt, Germany). Isopropanol, chloroform and acetonitrile were purchased from VWR International (Radnor, PA, USA).

### Locomotor activity

Variations in the locomotor activity were evaluated after treatment of the three ant species with either carvacrol (C) or thymol (T), or either a 3:1 (v/v) (Ct) or a 1:3 (v/v) (Tc) mixture of carvacrol/thymol. A 477 μg ml^-1^ solution of the above chemical and mixture was used in order to provide a final concentration of 0.1 ppm inside the Petri dish (9 cm internal diameter × 1.5 cm height). 20 μL of each solution were poured on a small paper disk (5 mm diameter) which was left in the centre of a Petri dish. Two controls were used in the bioassays: the first to assess the effect of the artificial condition (blank) and the second to evaluate the effect of the solvent (DMSO) on the ant movements.

Afterwards, 18 *F*. *cinerea*, 30 *M*. *scabrinodis* and 30 *T*. *caespitum* ants were randomly chosen among foraging workers. Due to their different size, three *F*. *cinerea* ants (6–7 mm each) and five workers in the case of *M*. *scabrinodis* and *T*. *caespitum* (around 4 mm each) were placed per each Petri dish and allowed to settle for 5 minutes. Ants of each species were tested simultaneously in six Petri dishes (4 treatments and 2 controls). Three colonies per ant species were assayed (N = 9) and a total of 234 workers were observed.

Protocols described by Hojo and colleagues [31] were followed to assess the ant locomotion. A bisecting line (i.e., one diameter) was drawn on the lid of each Petri plate. The number of times each ant crossed the solid line was recorded for 1 h by a video camera (Panasonic HC-4k) and measured by operators who were blind to experimental condition. Ants were not marked, thus crossings were counted irrespectively of the individual. No deviant behaviour (e.g., stay still) of any individual was observed during the bioassay.

### Aggression bioassays

Aggression bioassays were performed on *Myrmica* foraging workers of 3 colonies. All ants were marked on the thorax and allowed to recover before the aggression bioassay was carried out. Bioassays were performed as described by Csata *et al*. [32]. Briefly, two transparent plastic tubes (3 cm long) were joined and separated by a small piece of red plastic foil. One worker was placed per tube and after one minute the plastic foil was removed.

Individuals of the same colony were treated with either C, T, Ct or Tc. Ants were separated in a Petri dish each containing a single worker which was treated for 30 min with 0.1 ppm of the aforementioned compounds. Then bioassays were carried out testing non-nestmate (heterocolonial) ants with the following combinations, carvacrol vs carvacrol (CC), thymol vs thymol (TT), carvacrol/thymol vs carvacrol/thymol (Ct-Ct) and thymol/carvacrol vs thymol/carvacrol (Tc-Tc). Control tests were carried out using ants left in a Petri dish for 30 min without addition of compounds (CTRL). For each treatment, three tests using all possible combinations of the three colonies were replicated three times. Observations started with the first contact of the workers and lasted for three minutes. All behaviours were recorded, including biting, pulling, stinging, allogrooming and antennation. The latter two were considered as positive or neutral interactions, respectively, whilst the other behaviours were categorized as aggressive. The aggression index (AI) was calculated as the number of aggressive behaviours divided by the total number of interactions.

### Ant brain dissection and sample preparation

Sample preparation and the dissection of ant brains were performed following the protocols by Hojo *et al*. [31]. Briefly, two weeks after freezing at -25°C, ants were rapidly beheaded under a dissection microscope using micro-scissors. A small medial-lateral incision was made directly behind the mandibles and, in order to prevent contamination by retinal pigments, optic lobes were removed from the rest of the brain. Each sample contained two brains with each dissection time averaging less than 1 min. Brains were homogenized in 20 μl water solution containing 0.05% (v/v) formic acid and 1 ng 3,4-di-hydroxybenzylamine (DHBA) used as an internal standard. After vortex mixing, samples were centrifuged at 10,000 *g* for 20 min at 4°C. In order to remove undesirable compounds, a solution of isopropanol/chloroform (1:4, v/v) was directly added to the supernatant in a 1:1 (v/v) ratio. Samples were centrifuged at the same conditions described above. The aqueous layer obtained after centrifugation was immediately frozen on dry ice and stored at -80°C until injection into an HPLC-ESI-MS/MS (1200 HPLC, Agilent Technologies, Santa Clara, CA, USA) for amine quantification.

### Isolation and quantification of biogenic amines by HPLC-ESI-MS/MS

Biogenic amines (serotonin, dopamine and tyramine) and the internal standard DHBA were identified and quantified by liquid chromatography (1200 HPLC, Agilent Technologies, Santa Clara, CA, USA) equipped with a reverse phase Kinetex F5 Core-Shell LC Column (2,6 μm, 150 mm × 3.0 mm, 100 Å, Phenomenex, Torrance, CA, USA) which was kept at 40°C during chromatography. The binary solvent system was: (A) MilliQ H_2_O containing 0.1% (v/v) formic acid and (B) MeCN containing 0.1% (v/v) formic acid. The chromatographic separation was carried out at a constant flow rate (200 μl·min^-1^) with the following conditions: isocratic gradient at 3% B for the first 15 min, then 40% B in 5 min, then after 24 min B concentration was raised to 98%. The concentration of B was then maintained at 98% for 5 min. The initial mobile phase was re-established for 10 min before the next injection. The ionization of each amine was performed by electrospray ionization (ESI) operating in positive mode and tandem mass spectrometry analyses were performed with a 6330 Series Ion Trap LC-MS System (Agilent Technologies, USA). The flow rate of nitrogen was set at 325°C and 5.0 l·min^-1^, and the capillary voltage was set at 1.5 kV. Helium was used as a collision gas. Quantitative analyses were performed by Multiple Reaction Monitoring (MRM) by monitoring the fragmentation of quasi-molecular ions for serotonin (m/z: 177.1; 159.9), dopamine (m/z: 154.1; 137.0), tyramine (m/z: 138.0; 121.0) and DHBA (m/z: 140.0; 123.0). Quantification was performed by internal standard and external calibration curves with pure standards. Limit of Detections (LOD) and Limit of Quantification (LOQ) for each compound were determined as described in the complementary guideline of validation of analytical procedures [33] (see S1 Table).

### Statistical analysis

Kolmogorov–Smirnov tests were used to assess the data distribution. Aggression indices were compared using a linear mixed model (LMM) with the colony ID as a random factor and CC, TT, Ct-Ct, Tc-Tc, CTRL as fixed factors. For locomotor activity (LA) assays, data were analysed using a linear mixed model with cumulated number of crossings as a response variable, ant colony as a random intercept, and treatment (CTRL, DMSO, C, T, Ct, Tc) as fixed factors. Differences in the amount of each biogenic amine was tested by LMM using ant colony as a random factor and treatments (CTRL, DMSO, C, T, Ct, Tc) as fixed effect. Tukey’s honest significant difference (HSD) post hoc was used to test pairwise comparisons. Pearson’s coefficients were used to test the correlation between chemical and behavioural data. All statistics were performed using SPSS package ver. 25.

## Results

### Oregano terpenoids modulate ant locomotor activity

**Fig 1:**
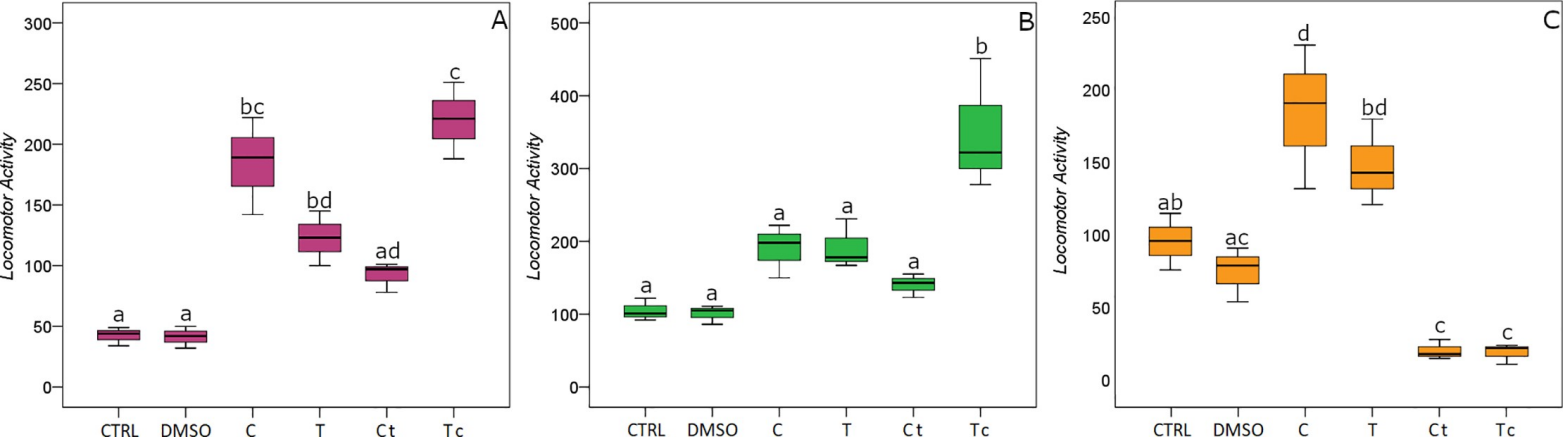
Ant locomotor activity. Data are expressed as the frequency of crossing the bisector traced on the Petri lid, taken as a proxy for worker locomotor activity affected by treatments (C: carvacrol; T: thymol; Ct: 3:1 (v/v) carvacrol/thymol; Tc: 1:3 (v/v) carvacrol/thymol) and controls (CTRL and DMSO) on *Formica cinerea* (A), *Tetramorium caespitum* (B) and *Myrmica scabrinodis* (C). Boxplots show median, quartile, maximum and minimum values; different letters indicate significant differences (Tukey’s HSD post hoc, P < 0.05; S2 Table).

Sublethal concentrations of carvacrol and thymol triggered a significant variation in the locomotor activity (LA) of the three ant species under study (Fig 1).

Single or mixed compounds increased LA in *F*. *cinerea* with respect to controls (Fig 1A; F_18,5_ = 28.641; P < 0.001). Movements were enhanced especially when ants were treated with Tc and, to a lesser extent, with C and T. Although increased, LA of workers exposed to a blend with a larger amount of Ct did not vary significantly from controls (S2 Table). Differences in the LA were also found in treated and untreated *T*. *caespitum* workers (Fig 1B; F_18,5_ = 13.658; P < 0.001). Overall, treatments elicited an increase in ant movements, but this variation was significant only in response to Tc. A different response was found when LA was assayed in *M*. *scabrinodis*. Treatments affected the movements of *M*. *scabrinodis* (Fig 1C; F_18,5_ = 19.268; P < 0.001) and exposure to C and T induced an LA increase in workers. In contrast, both Ct and Tc caused a significant decrease in ant movements.

### Oregano terpenoids modulate brain biogenic amine content

**Fig 2:**
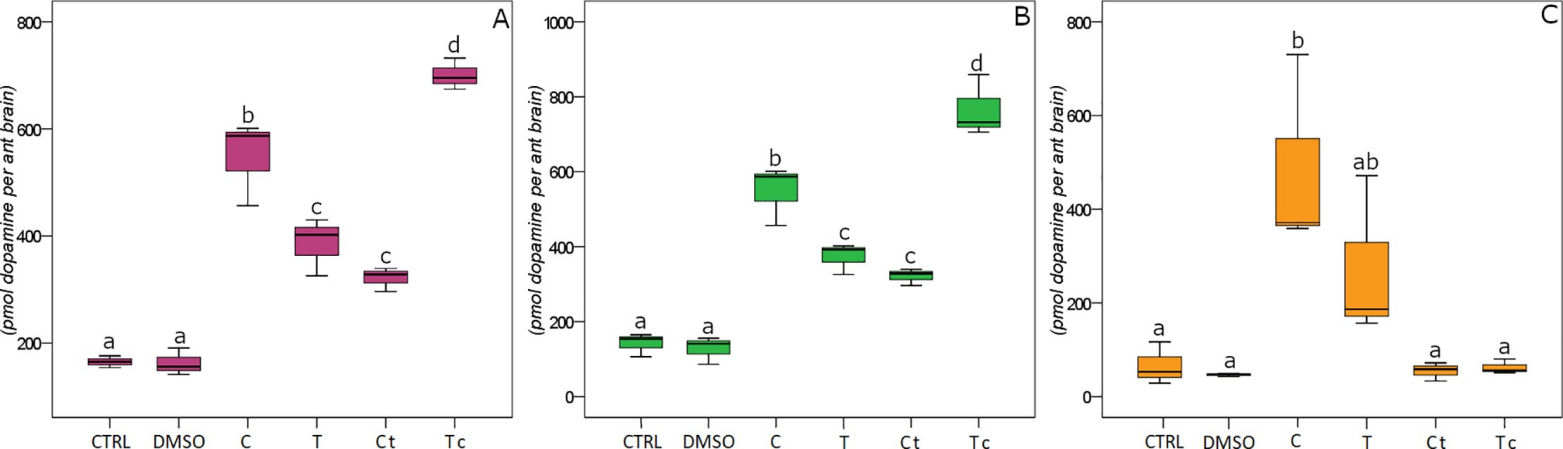
Dopamine content. Effects of treatments (C: carvacrol; T: thymol; Ct: 3:1 (v/v) carvacrol/thymol; Tc: 1:3 (v/v) carvacrol/thymol) and controls (CTRL and DMSO) on dopamine contents in ant brains of *Formica cinerea* (A), *Tetramorium caespitum* (B) and *Myrmica scabrinodis* (C). Values are expressed as pmol dopamine per ant brain. Boxplots show median, quartile, maximum and minimum values; different letters indicate significant differences (Tukey’s HSD post hoc, p < 0.05; S3 Table).

Treatments with C and T significantly affected the brain content of dopamine in *F*. *cinerea* (Fig 2A: F_18,5_ = 63.744, P < 0.001), *T*. *caespitum* (Fig 2B: F_18,5_ = 58.104, P<0.001) and *M*. *scabrinodis* (Fig 2C: F_18,5_ = 6.500, P = 0.006).

**Fig 3:**
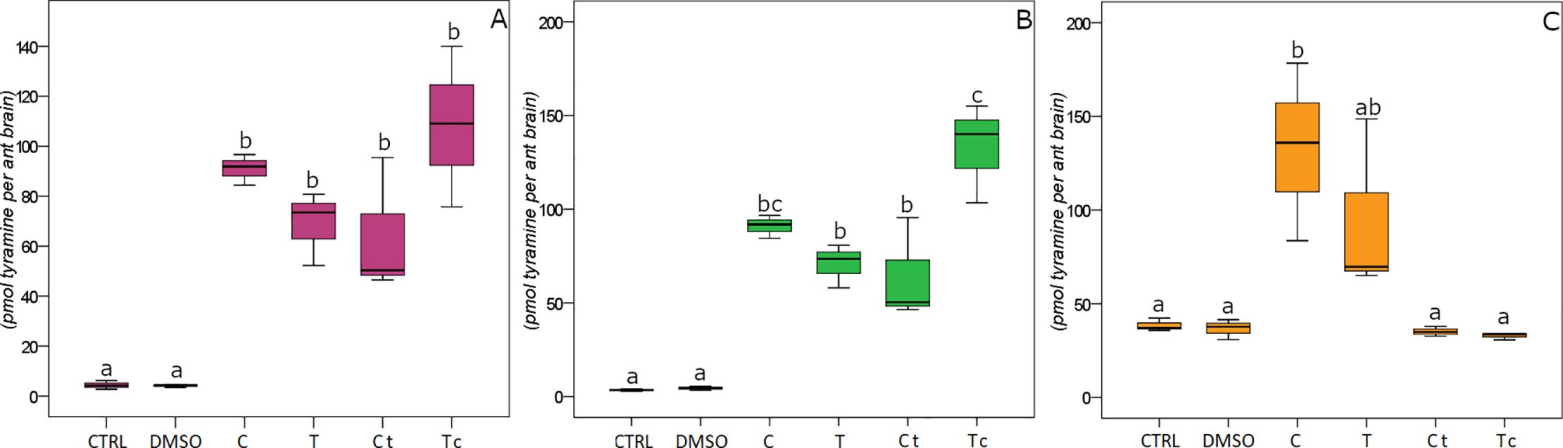
Tyramine content. Effects of treatments (C: carvacrol; T: thymol; Ct: 3:1 (v/v) carvacrol/thymol; Tc: 1:3 (v/v) carvacrol/thymol) and controls (CTRL and DMSO) on tyramine contents in ant brains of *Formica cinerea* (A), *Tetramorium caespitum* (B) and *Myrmica scabrinodis* (C). Values are expressed as pmol tyramine per ant brain. Boxplots show median, quartile, maximum and minimum values; different letters indicate significant differences (Tukey’s HSD post hoc, p < 0.05; S3 Table).

Tyramine brain content was also significantly affected by Oregano terpenoids in *F*. *cinerea* (Fig 3A: F_18,5_ = 15.799, P < 0.001), *T*. *caespitum* (Fig 3B: F_18,5_ = 27.315, P < 0.001) and *M*. *scabrinodis* (Fig 3C: F_18,5_ = 5.963 P = 0.008).

Irrespectively of the species, brain dopamine and tyramine contents were similarly affected by treatments, as they both increase or decrease in response to the same compound (all ant species Pearson correlation; ρ = 0.97, P < 0.001).

In *F*. *cinerea*, the highest dopamine content was found after Tc treatment. C and, to a lesser extent T and Ct, triggered increased dopamine levels (Fig 2A; S3 Table). A similar pattern was found for tyramine level, which was correlated to dopamine content (Pearson correlation; ρ = 0.96, P < 0.001). Significant changes in tyramine content were only reported between treatments and controls (Fig 3A; S3 Table).

In *T*. *caespitum*, C and Tc caused the largest increase in the dopamine and tyramine levels (Figs 2B and 3B), which showed a significant correlation (Pearson correlation ρ = 0.98, P < 0.001). T and Ct triggered a lower but significant effect, with respect to controls (Fig 2B; S3 Table).

In *M*. *scabrinodis*, the levels of brain dopamine and tyramine varied consistently (Pearson correlation. ρ = 0.99, P < 0.001). With respect to controls, C and T triggered an increase of both dopamine and tyramine (Figs 2C and 3C; S3 Table). Although not significant, an average decrease in tyramine is elicited by exposure to the two blends (Fig 3C; S3 Table).

The locomotor activity of all ant species was strongly correlated with changes in the dopamine (Pearson correlation; ρ = 0.94, P < 0.001) and tyramine (Pearson correlation; ρ = 0.91, P < 0.001) levels.

In contrast to dopamine and tyramine, no significant variation in the serotonin brain content was reported in all ant species (S1 Fig).

### Oregano terpenoids induce M. scabrinodis aggression

**Fig 4:**
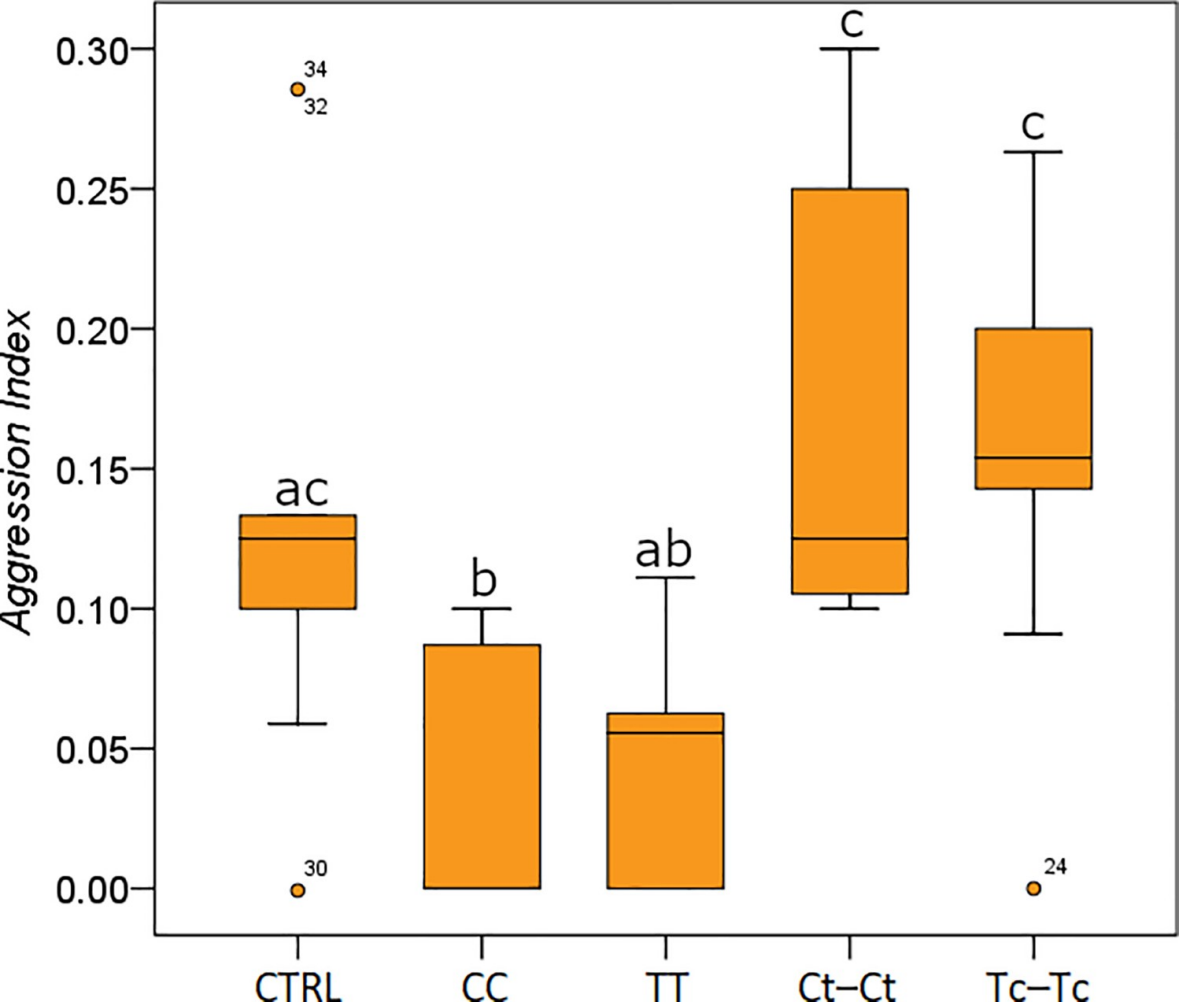
Aggression index. Aggression index between heterocolonial *M*. *scabrinodis* workers treated with different compounds or pure air (CTRL: non-treated; CC: carvacrol *vs*. carvacrol; TT: thymol *vs*. thymol; Ct-Ct: 3:1 (v/v) carvacrol/thymol *vs*. 3:1 (v/v) carvacrol/thymol, and Tc-Tc 1:3 (v/v) carvacrol/thymol *vs*. 1:3 (v/v) carvacrol/thymol). Data are expressed as number of aggressive behaviours on the total number of interaction (see methods for details). Boxplots show median, quartile, maximum and minimum values; different letters indicate significant differences (Tukey’s HSD post hoc, p < 0.05; S4 Table).

Aggression behaviour was assessed in *M*. *scabrinodis* workers only, because this species is directly involved in the associations with *O*. *vulgare* plants and in the interaction with the *Maculinea* butterfly parasite. The level of aggression varied with exposure to different treatments (Fig 4: F_45,4_ = 8.031, P = 0.007).

Aggression was significantly lower when heterocolonial ants were treated with CC, whilst TT caused a reduction in the antagonist behaviour, which was not significantly different from the control tests (Fig 4; S4 Table). Exposure to Ct-Ct and Tc-Tc significantly increased the aggression of *M*. *scabrinodis*, when compared to C and T treatments (Fig 4; S4 Table). Changes in the worker aggression were negatively correlated with dopamine (Pearson correlation ρ = -0.93, P < 0.001) and tyramine (Pearson correlation ρ = -0.90, P < 0.001).

## Discussion

The results of this work show that C and T modulate the locomotor activity of some ant species. Treatments with either C, T, or mixed solutions differently affected ant movements. *F*. *cinerea* and *T*. *caespitum* workers generally increased their propensity to move in response to Tc, whereas the increase in *M*. *scabrinodis* movements only occurred when either C or T were administered alone.

In general, an increased locomotor activity is used as a proxy for ant escaping behaviour, whereas its reduction indicates an enhanced partner fidelity [31]. The dissimilar response of ant species to *O*. *vulgare* terpenoids might be linked to their different success in detoxifying these plant volatiles. Both T and C can be detrimental for insect [34] and C has been uses as insecticide [34–36], fungicidal [34,37] and acaricide [35], although the mechanism of its action is still unclear [37–39]. *Myrmica* ants are able to adapt and survive to environmental doses of C by upregulating genes (e.g., acetylcholinesterase, glutathione S transferase and CYP4509E2) whose products bind and detoxify this monoterpene, by making the colonies more resistant than other common ant species [28]. On the contrary, *F*. *cinerea* and *T*. *caespitum* did not show any significant upregulation of detoxifying genes when exposed to C and died soon after the treatment [28]. Hence, the increased locomotor activity here reported for *F*. *cinerea* and *T*. *caespitum* upon treatment with C and T can be considered as a countermeasure to enhance their chances to survive.

Interestingly, a reduction in the locomotor activity is observed only in *M*. *scabrinodis* as a response to terpenoid blends (i.e., Ct, Tc). In natural environments, T is always released by *O*. *vulgare* [40] whilst C primarily occurs as a response to the ant-plant interaction [28]. Therefore, blends of the two terpenoids are mainly emitted when oregano plants respond to the presence of ants. The release of C and T mixtures is more than a generic deterrent signal [41] causing the dispel of some ant species and act as chemical cue manipulating the behavior of *Myrmica* ant. C and T blends not only decrease the *M*. *scabrinodis* locomotor ability (Fig 1), but also modulate and enhance another crucial behavioural trait, such as the worker aggressiveness (Fig 4).

The decrease in the ant propensity to move is consistent with field data demonstrating that *Myrmica* workers forage closed to *O*. *vulgare* plants more frequently than other ant species [28]. The co-occurrence of *Myrmica* and oregano plants has been previously explained [28] as a benefit for *Myrmica* ants to colonise an enemy-free space [42]. However the exploitation of the ground underneath the plants rises the risk of *Myrmica* colonies to be parasitized by *Maculinea* larvae, overall reducing the evolutionary advantage of colonising this niche [28]. Our results provide hints on how these complex dynamics might persist, suggesting that *Myrmica* ants are indeed manipulated (i.e., no net evolutionary advantage is expected) and workers are induced by the plant volatiles to stay and patrol the oregano surroundings [13,43].

On the plant side, the ability to attract a species which is not phytophagous [44] and to increase its chemical aggressiveness at the cost of a slight variation in the volatile emission could represent an advantageous strategy to get further protection against herbivores. There are several associations where ant pugnacity is exploited by plants as a defence against enemies (e.g. herbivores, encroaching vegetation) [45,46]. Recently, it has been demonstrated that the plant-ant interaction *Acacia*-*Pseudomyrmex* is not an entirely mutualistic association, but definitely an example of “partner manipulation” [47]. Through the manipulation of the worker digestive activity the *Acacia* makes the ants strictly dependent, thus enhancing partner fidelity and protection.

A similar scenario has been described by Hojo and co-workers [31] studying a lycaenid larva and its “supposed” mutualistic associated ants. The butterfly larva secretes manipulative drugs which lower the attendance ant locomotor activity and enforce ant aggressive behaviour through dopaminergic regulation.

The role of dopamine in modulating insect movements and aggression has been shown in *Drosophila melanogaster* [17,48] and some monoamines are also known to be involved in the response to biotic and abiotic stress, including the variation of environment temperatures [49], population density [50] and food and water availability [51]. Biogenic amines, like dopamine, may function as neurotransmitters, neuromodulators, or neurohormones; however, studies showing a direct causal role of these molecules in mediating the behavioural plasticity are rare [19], mainly performed on invertebrates [20], and especially in social insects [52].

A wide variation of biogenic amine effects has been reported [20], showing how complex is the neurochemical mechanisms underlying social behaviours in ants and making any generalisation difficult. In contrast, the correlation between the biogenic amine levels and the ant locomotor behaviour we found is consistent throughout the three ant species. Our results show that dopamine levels, which are known to be directly correlated to the complexity of behavioral repertoire [53,54], and the tyramine content rise as the locomotor ability increases. This correlation suggests a widespread (across the ant species) and crucial role of dopamine and tyramine in modulating ant moving propensity, whereas serotonin appears not to be involved. Furthermore, our findings concur with the general knowledge [20,55] that, unlike other Hymenoptera, the ant aggression is not modulated by the serotonergic system.

Although further fieldwork experiments are needed and the mechanism through which plant volatiles can modulate biogenic amines content in ant brains is not yet clear, our study provides evidence of an interspecific behavioural manipulation through neurogenic dopamine and tyramine regulation.

## Conclusions

Ants represent a good model system for neuroethological studies because of their relatively simple neural architecture. Our work provides new insights on the role of plant terpenoids in dopamine and tyramine modulation for the regulation of the locomotor activity and aggressiveness of ant workers, supporting a direct function of biogenic amines in the control of behaviour and colony organization. In the *Maculinea*-*Myrmica*-*Origanum* system the two plant monoterpenes, C and T, play crucial roles by modulating ant behaviour, through aminergic regulation, and fostering bi-level interspecific associations. Understanding how the flow of information among distinct biological levels works will be a fundamental challenge to better understand the interplay between different trophic levels [56,57].

It is increasingly clear that several interactions which have been considered to be mutualistic in the past, are nowadays revised as potentially parasitic because the ant partner is not receiving an actual reward but it is rather manipulated [31, 47]. In the future, the identification of specific neural networks on which biogenic amines act and the identification of genes involved in the development of these neurons, along with ethological studies, will be pivotal to understand how behavioural manipulation is widespread and actually achieved in plant-ant interactions.

## Supporting information

S1 Table Linear regression, coefficient of determination (R^2^), limit of detection (LOD) and of quantification (LOQ) of biogenic amines. (DOCX)

S2 Table Tukey’s HSD post hoc differences in ant locomotor activity. *P<0.05; **P<0.01; ***P<0.001
(DOCX)

S3 Table Tukey’s HSD post hoc differences in ant brain dopamine and tyramine contents. *P<0.05; **P<0.01; ***P<0.001
(DOCX)

S4 Table Tukey’s HSD post hoc differences in aggression index between heterocolonial *Myrmica* workers. *P<0.05; **P<0.01.
(DOCX)

S1 Fig Serotonin content. Effects of treatments (C: carvacrol; T: thymol; Ct: 3:1 (v/v) carvacrol/thymol; Tc: 1:3 (v/v) carvacrol/thymol) and controls (CTRL and DMSO) on serotonin contents in ant brains of *Formica cinerea* (A), *Tetramorium caespitum* (B) and *Myrmica scabrinodis* (C). Values are expressed as pmol serotonin per ant brain. Boxplots show median, quartile, maximum and minimum values. No significant difference in the level of serotonin was found among the treatments.
(DOCX)
