# Correction: *Origanum vulgare* terpenoids modulate *Myrmica scabrinodis* brain biogenic amines and ant behaviour

**DOI:** 10.1371/journal.pone.0211749

**Published:** 2019-01-29

**Authors:** Giuseppe Mannino, Gholamreza Abdi, Massimo Emilio Maffei, Francesca Barbero

One of the affiliations for the second author is not indicated. Gholamreza Abdi is affiliated with the Department of Life Sciences and Systems Biology, Innovation Centre, University of Turin, Turin, Italy and the Department of Biotechnology, Persian Gulf Research Institute, Persian Gulf University, Bushehr, Iran.
