# Supplementary material for: Origanum vulgare terpenoids modulate Myrmica scabrinodis brain biogenic amines and ant behaviour
Source: PLoS One. 2018 Dec 26;13(12):e0209047. doi: 10.1371/journal.pone.0209047 (PMC6306168; doi:10.1371/journal.pone.0209047)
Supplement: S1 Table — (DOCX) [file pone.0209047.s001.docx]

**S1 Table**. Linear regression, coefficient of determination (R^2^), limit of detection (LOD) and of quantification (LOQ) of biogenic amines.

|  | **Regression Equation** | **R^2^** | **LOD** *(pmol)* | **LOQ** *(pmol)* |
| --- | --- | --- | --- | --- |
| Tyramine | y =8.62e^6^x-2.69e^4^ | 0.9968 | 4.4896 | 13.6051 |
| DHBA | y =8.28e^5^x+5.81e^4^ | 0.9965 | 2.5374 | 7.6891 |
| Dopamine | y =7.32e^6^x+6.04e^4^ | 0.9917 | 2.4971 | 7.5672 |
| Serotonin | y =6.49e^6^x-3.90e^4^ | 0.9839 | 2.9483 | 8.9344 |
