# Supplementary material for: Origanum vulgare terpenoids modulate Myrmica scabrinodis brain biogenic amines and ant behaviour
Source: PLoS One. 2018 Dec 26;13(12):e0209047. doi: 10.1371/journal.pone.0209047 (PMC6306168; doi:10.1371/journal.pone.0209047)
Supplement: S2 Table — *P<0.05; **P<0.01; ***P<0.001 (DOCX) [file pone.0209047.s002.docx]

**S2 Table**. Tukey’s HSD post hoc differences in ant locomotor activity. *P<0.05; **P<0.01; ***P<0.001

|  | | **Locomotor Activity** | | |
| --- | --- | --- | --- | --- |
|  |  | *F. cinerea* | *T. caespitum* | *M. scabrinodis* |
| CTRL | DMSO | 1.00 | 4.33 | 2.00 |
|  | C | -142.01*** | -85.00 | -103.67* |
|  | T | -80.33* | -87.00 | -72.67 |
|  | C/T | -49.67 | -35.33 | 19.67 |
|  | T/C | -177.67*** | -245.33*** | 14.02 |
| DMSO | C | -143*** | -89.33 | -105.67* |
|  | T | -81.33* | -91.33 | -74.67 |
|  | C/T | -50.67 | -39.67 | 17.67 |
|  | T/C | -178.67*** | -249.67*** | 12.01 |
| C | T | 61.67 | -2.00 | 31.01 |
|  | C/T | 92.33** | 49.67 | 123.33* |
|  | T/C | -35.67 | -160.33** | 117.67* |
| T | C/T | 30.67 | 51.67 | 92.33 |
|  | T/C | -97.33** | -158.33** | 86.67 |
| C/T | T/C | -128.00*** | -210.00** | -5.67 |
