# Supplementary material for: Origanum vulgare terpenoids modulate Myrmica scabrinodis brain biogenic amines and ant behaviour
Source: PLoS One. 2018 Dec 26;13(12):e0209047. doi: 10.1371/journal.pone.0209047 (PMC6306168; doi:10.1371/journal.pone.0209047)
Supplement: S3 Table — *P<0.05; **P<0.01; ***P<0.001 (DOCX) [file pone.0209047.s003.docx]

**S3 Table**. Tukey’s HSD post hoc differences in ant brain dopamine and tyramine contents. *P<0.05; **P<0.01; ***P<0.001

|  | | **Dopamine** | | | **Tyramine** | | |
| --- | --- | --- | --- | --- | --- | --- | --- |
|  |  | *F. cinerea* | *T. caespitum* | *M. scabrinodis* | *F. cinerea* | *T. caespitum* | *M. scabrinodis* |
| CTRL | DMSO | 2.391 | 14.013 | 19.578 | 0.291 | -1.025 | 1.751 |
|  | C | -383.024*** | -406.197*** | -420.568** | -86.608** | -87.519*** | -94.207* |
|  | T | -220.871*** | -231.517** | -205.556 | -64.457* | -67.276** | -56.037 |
|  | C/T | -156.337** | -179.509* | 11.501 | -59.709* | -60.620** | 3.228 |
|  | T/C | -535.459*** | -623.886*** | 3.809 | -103.892*** | -129.378*** | 5.562 |
| DMSO | C | -385.415*** | -420.21*** | -440.147** | -86.900** | -86.493*** | -95.957* |
|  | T | -223.261*** | -245.53** | -225.134 | -64.748* | -66.250** | -57.787 |
|  | C/T | -158.728** | -193.523** | -8.076 | -60.001* | -59.594** | 1.478 |
|  | T/C | -537.849*** | -637.899*** | -15.768 | -104.183*** | -128.352*** | 3.812 |
| C | T | 162.153** | 174.679* | 215.013 | 22.151 | 20.243 | 38.17 |
|  | C/T | 226.687*** | 226.687** | 432.070** | 26.899 | 26.899 | 97.436** |
|  | T/C | -152.434* | -217.689** | 424.378** | -17.283 | -41.858 | 99.770** |
| T | C/T | 64.533 | 52.007 | 217.057 | 4.747 | 6.655 | 59.265 |
|  | T/C | -314.588*** | -392.368*** | 209.365 | -39.434 | -62.101** | 61.599 |
| C/T | T/C | -379.121*** | -444.376*** | -7.691 | -44.182 | -68.757** | 2.334 |
