# Supplementary material for: Origanum vulgare terpenoids modulate Myrmica scabrinodis brain biogenic amines and ant behaviour
Source: PLoS One. 2018 Dec 26;13(12):e0209047. doi: 10.1371/journal.pone.0209047 (PMC6306168; doi:10.1371/journal.pone.0209047)
Supplement: S4 Table — *P<0.05; **P<0.01. (DOCX) [file pone.0209047.s004.docx]

**S4 Table**. Tukey’s HSD post hoc differences in aggression index between heterocolonial *Myrmica* workers. *P<0.05; **P<0.01.

|  |  | **Aggression Index** |
| --- | --- | --- |
|  | | *M. scabrinodis* |
| CTRL | CC | 0.095* |
|  | TT | 0.086 |
|  | CT_CT | -0.035 |
|  | TC_TC | -0.017 |
| CC | TT | -0.009 |
|  | CT_CT | -0.13** |
|  | TC_TC | -0.113* |
| TT | CT_CT | -0.121** |
|  | TC_TC | -0.104* |
| CT_CT | TC_TC | 0.017 |
