# Supplementary material for: Origanum vulgare terpenoids modulate Myrmica scabrinodis brain biogenic amines and ant behaviour
Source: PLoS One. 2018 Dec 26;13(12):e0209047. doi: 10.1371/journal.pone.0209047 (PMC6306168; doi:10.1371/journal.pone.0209047)

**S1 Fig**. **Serotonin Content**. Effects of treatments (C: carvacrol; T: thymol; Ct: 3:1 (v/v) carvacrol/thymol; Tc: 1:3 (v/v) carvacrol/thymol) and controls (CTRL and DMSO) on serotonin contents in ant brains of *Formica cinerea* (A), *Tetramorium caespitum* (B) and *Myrmica scabrinodis* (C). Values are expressed as pmol serotonin per ant brain. Boxplots show median, quartile, maximum and minimum values. No significant difference in the level of serotonin was found among the treatments.


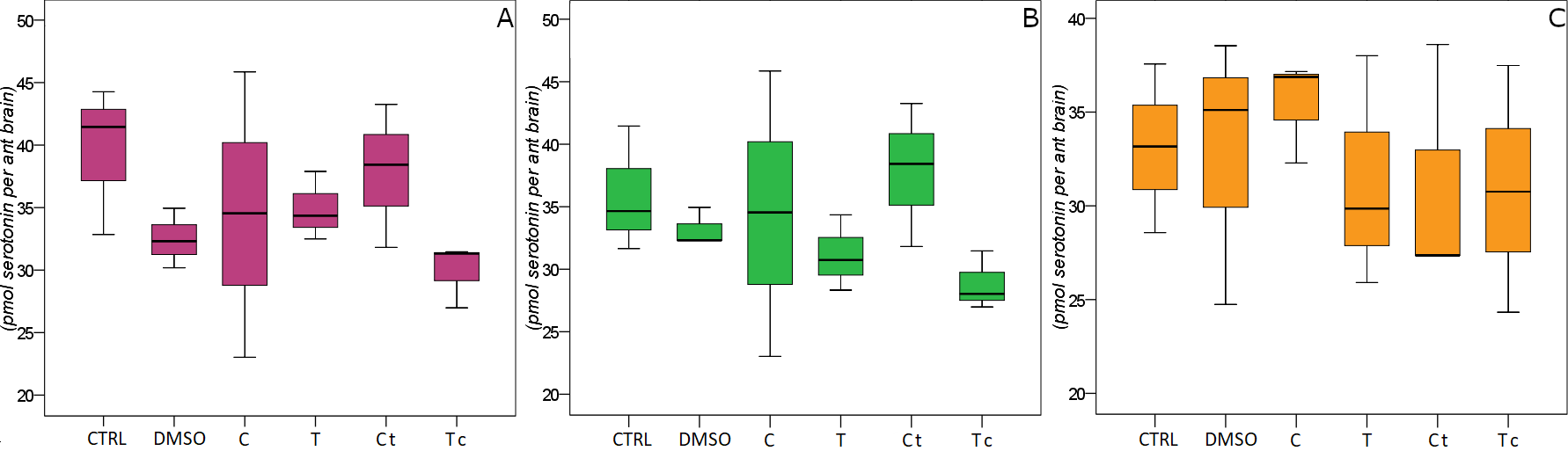

Supplement: S1 Fig — Effects of treatments (C: carvacrol; T: thymol; Ct: 3:1 (v/v) carvacrol/thymol; Tc: 1:3 (v/v) carvacrol/thymol) and controls (CTRL and DMSO) on serotonin contents in ant brains of Formica cinerea (A), Tetramorium caespitum (B) and Myrmica scabrinodis (C). Values are expressed as pmol serotonin per ant brain. Boxplots show median, quartile, maximum and minimum values. No significant difference in the level of serotonin was found among the treatments. (DOCX) [file pone.0209047.s005.docx]
